# Flow patterns in circular fish tanks and its relations with flow rate and nozzle features

**DOI:** 10.1038/s41598-022-17186-z

**Published:** 2022-07-28

**Authors:** El-Sayed Khater, Samir Ali, Wael Abbas, Osama Morsy

**Affiliations:** 1 Agricultural and Biosystems Engineering Department, Faculty of Agriculture, Benha University, P.O. Box 13736, Moshtohor, Toukh, Kalubia Egypt; 2 Basic and Applied Science Department, College of Engineering and Technology, Arab Academy for Science and Technology and Maritime Transport (AASTMT), P.O. Box 2033, Cairo, Egypt

**Keywords:** Ecology, Environmental sciences, Engineering

## Abstract

The main aim of this research is to study the effect of flow rate, diameter and number of nozzles on the rotational velocity, impulse force and average velocity of water in circular fish tanks. The results indicated that, the rotational velocity increases with increasing flow rates from 5 to 75 m^3^ h^−1^ by using 10 and 15 mm nozzles diameter with nozzles number of 5, 10, 15, 20, 25 and 30. The same results were also obtained when 20 and 25 mm nozzle diameter with nozzles number of 3, 6, 9, 12 and 15 were used. The impulse force of water decreases with increasing nozzle diameter from 10.0 to 15.0 mm with 5, 10, 15, 20, 25 and 30 nozzles and from 20.0 to 25.0 mm with 3, 6, 9, 12 and 15 nozzles. When the nozzle diameter increased from 10 to 25 mm the impulse force of water decreased with 15 nozzles. The average velocity of water decreases with increasing nozzles diameter from 10.0 to 15.0 mm with 5, 10, 15, 20, 25 and 30 nozzles and from 20.0 to 25.0 mm with 3, 6, 9, 12 and 15 nozzles. When the nozzle diameter increased from 10 to 25 mm, the average velocity ranged from 1.07 to 48.76 cm s^−1^ for all treatments under study.

## Introduction

The suitable conditions of rearing aquatic organisms could be designed the aquaculture system. Aquaculture compatible with environmental restrictions and with other important economic activities would be made such as tourism or fishing, tanks by guarantee fish welfare, resource consumption minimization (feed, oxygen, energy) and decreasing labor costs, which cause less environmental effect and use the smallest area possible^1^.

Due to biosolids sedimentation on the bottom of tanks, water velocity in the tanks should be controlled because these wastes particles cause pollution hypoxic conditions as well as consume oxygen. To control water velocity, the inlet and outlet design should be design carefully to improve the general flow conditions inside the tanks^2^. The suitable water velocity ranged from 3 to 40 cm s^−1^ according to the physical properties of the biosolids. The presence of fish in the tanks helps too much in self-cleaning properties especially in the circular tanks^3,4^.

It is easy to adjust the tank parameters and configuration, if we can maintain the proper water velocity in tanks which affect the general health, muscle tone and respiration, which differs according to size and species of fish. The swimming speeds lower than the higher spontaneous activity. Meanwhile at speeds higher than optimal, swimming becomes unsustainable and stressful^5^. Also, anaerobic metabolism will increase lactate levels which creates an oxygen debt and cause fatigue^6^ The rotational velocity in a fish tank, which implicitly depends on inlet flow rate and hence the impulse force plays an important role in creating a healthy rearing environment. Flow pattern and turbulence in the fish tank are primarily affected by the inlet and outlet properties^7^.

Besides, the proper velocity in tanks, velocities distribution is very important. Non-uniformity distribution of water velocities cause less efficient use of space available, because fish avoid high velocities areas and lower dissolved oxygen and higher metabolite concentrations^8^. Distribution uniformity coefficient of velocities in fish tanks has been studied by many researchers. They found that the analysis of distribution uniformity needs both global assessment of average velocity and details of flow pattern analysis to determine the effect of design parameters on the homogeneity of velocity in fish tanks^9,10^.

Fish behavior and activities affected by tank hydrodynamics which is affected by inlet and outlet configurations and degree of fish swimming activity^1,11^. Tank hydrodynamics can produce heterogenous conditions by inducing fish to distribute heterogeneous conditions by inducing fish to distribute heterogeneously throughout the fish tanks^12^.

Improving fish welfare and reducing stress levels are controlled by tank design parameters. Homogenous water quality helps to use the advantages of rearing volume, water flow and oxygen added to water. Circular tank are commonly used in fish farming because it provide more steady flow patterns, more homogeneous distribution of dissolved oxygen and metabolites and better self-cleaning properties^9,13,14^. In order to maintain water velocity and flow rate that are suitable for rearing fish in tanks, therefore, the main objective of this work is to study the effect of flow rates, diameters and numbers of nozzles on the rotational velocity, impulse force and average velocity in the fish tanks.

## Materials and methods

The experiment was carried out at Agricultural and Bio-Systems Engineering Department, Faculty of Agriculture Moshtohor, Benha University, Egypt during of the 2018 season to choose the number and diameter orifice entering the water to the fish tank in order to maintain the rotational velocity of water suitable for fish of water in circular fish tanks.

### Materials

#### System description

**Figure 1:**
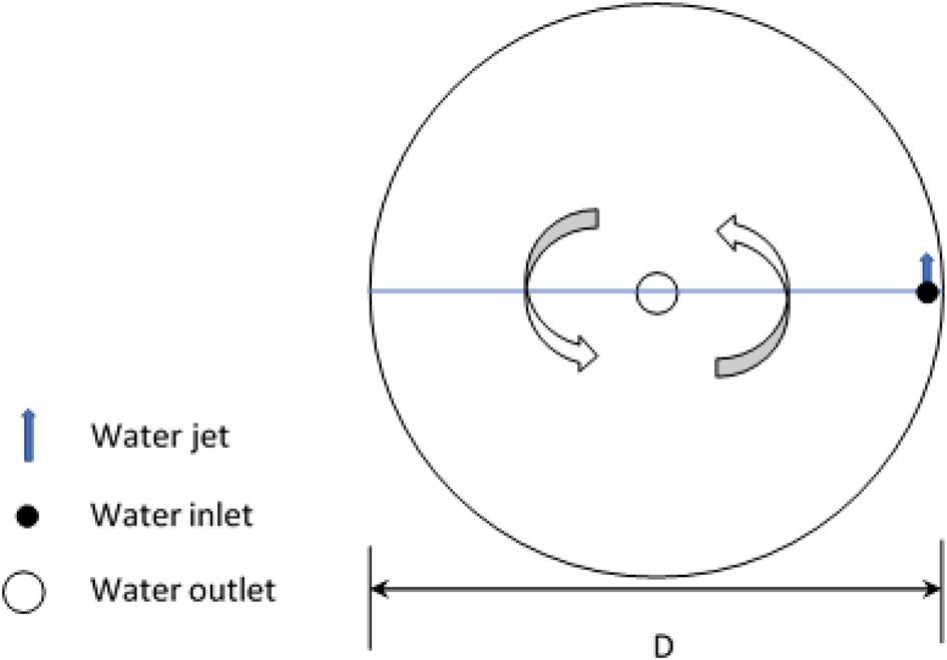
Fish tank.

**Figure 2:**
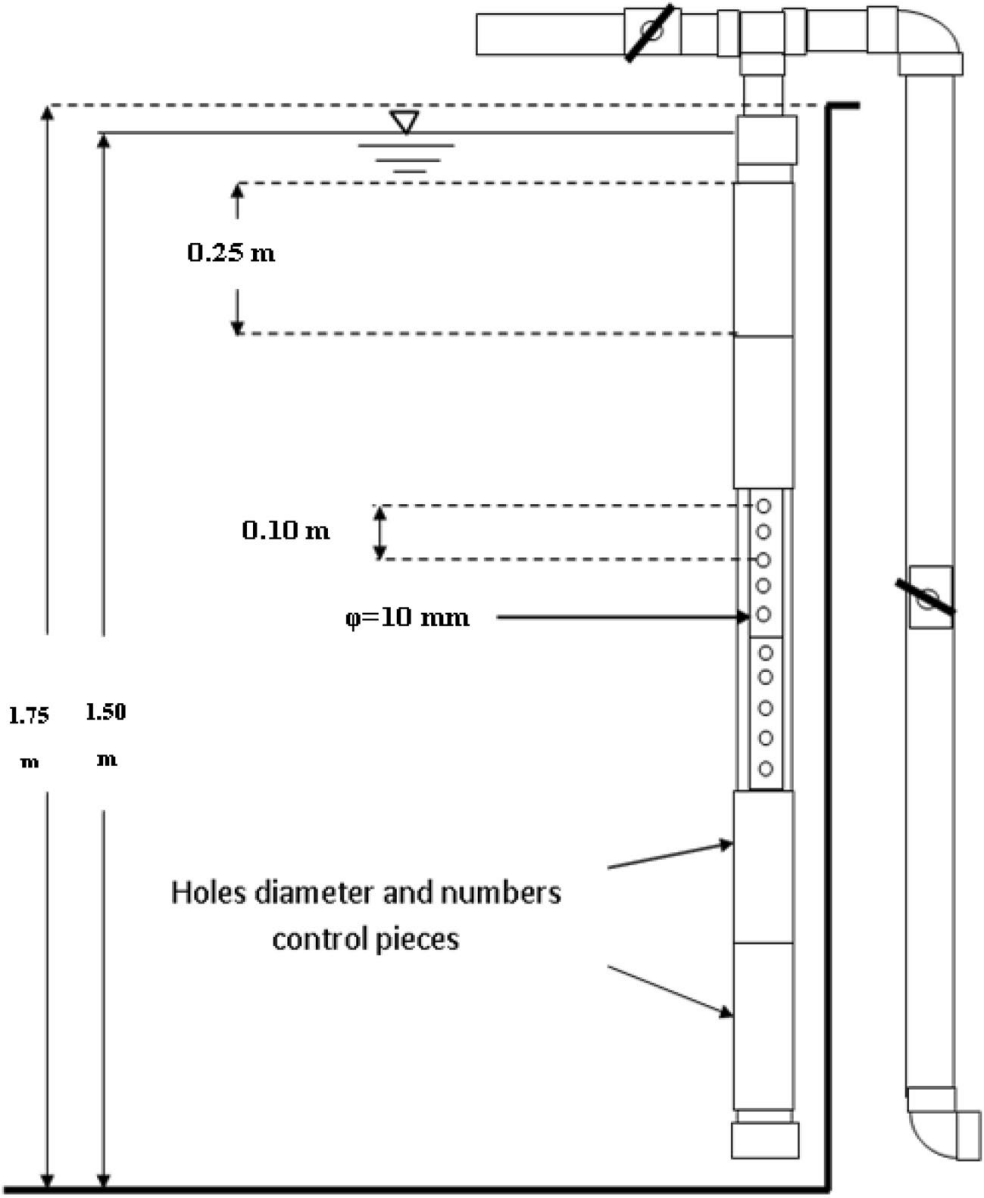
Submerged water jet.

The experiment was conducted in a circular tank made of concrete which was used for fish culture. Its diameter is 10 m and has a depth of 1.75 m. The level of water was controlled by standpipe to maintain a water depth of 1.50 m as shown in Figs. 1 and 2.

The rotational flow in the tank is created by the action of submerged water jets directed perpendicularly to the tank wall as shown in Fig. 1^15^.

A pipe of 110 mm diameter was used to have the holes of water jets, where, it is drilled on its four sides: first side is drilled at 5 cm with a diameter of 10 mm, the second side was drilled every 5 cm with hole diameter of 15 mm, third side was drilled each 10 cm with hole of 20 mm diameter and the fourth side is drilled at 10 cm spacing with holes with 25 mm diameter. To control the water jet flow, a slid cover was installed on the perforated pipe to obtain the required diameter and numbers of nozzles as show in Table 1.

**Table 1:** Nozzle diameters and numbers.

Nozzle diameters (mm)	Nozzle numbers (no.)
10	5, 10, 15, 20, 25 and 30
15	5, 10, 15, 20, 25 and 30
20	3, 6, 9, 12 and 15
25	3, 6, 9, 12 and 15

### Methods

The experiment was devoted to study the effect of water flow rate, nozzle diameters and nozzle numbers at jet angles of 90°. The experimental treatments were:

1. Six water flow rates (5.0, 15.0, 30.0, 45.0, 60.0 and 75.0 m^3^ h^−1^).
2. Four orifice diameters (10, 15, 20 and 25 mm).
3. Orifice numbers according to Table 1.

The number of nozzle and nozzles diameter were controlled through some pieces of 110 mm pipe cracked longitudinally as shown in Fig. 1. Temperature was kept at 28.0 ± 1.3 °C and salinity was 854 ppm.

### Measurements

Rotational velocity was measured directly in the fish tank by flow meter (Model FLO-MATE 2000—Range—0.15 to 6.0 m s^−1^—Accuracy ± 2%, USA). Rotational velocities were measured at 4 different locations distributed on circumference of the circular tank (at 10 cm from water jet at 0.75 m water depth, 7.85, 15.70 and 23.55 m, respectively). The average of these readings was taken.

Calculation of the impulse force of water in the tank was done using the formula below as stated by^16^:

1 $$ {\text{F}}_{{\text{i}}} = \rho Q\left( {V_{in} - V1} \right) $$

where F_i_ is the impulse force, N, ρ is the water density, kg m^−3^, *Q* is the discharge, m^3^ s^−1^, V_1_ the mean circulated velocity, m s^−1^, V_in_ the inlet water velocity, m s^−1^.

Calculation of the average velocity of water in the tank was done using the formula below as stated by^17^:

2 $$ {\text{V}}_{{{\text{avg}}}} = \sqrt {\frac{{2QV_{in} }}{{A \cdot C_{t} }}} $$

where V_avg_ is the average velocity, m s^−1^, A is the wetted area (tank floor area + round wall area), m^2^, C_t_ is the resistance coefficient of the tank (0.08) according to^16^.

## Results and discussion

### Rotational velocity

**Figure 3:**
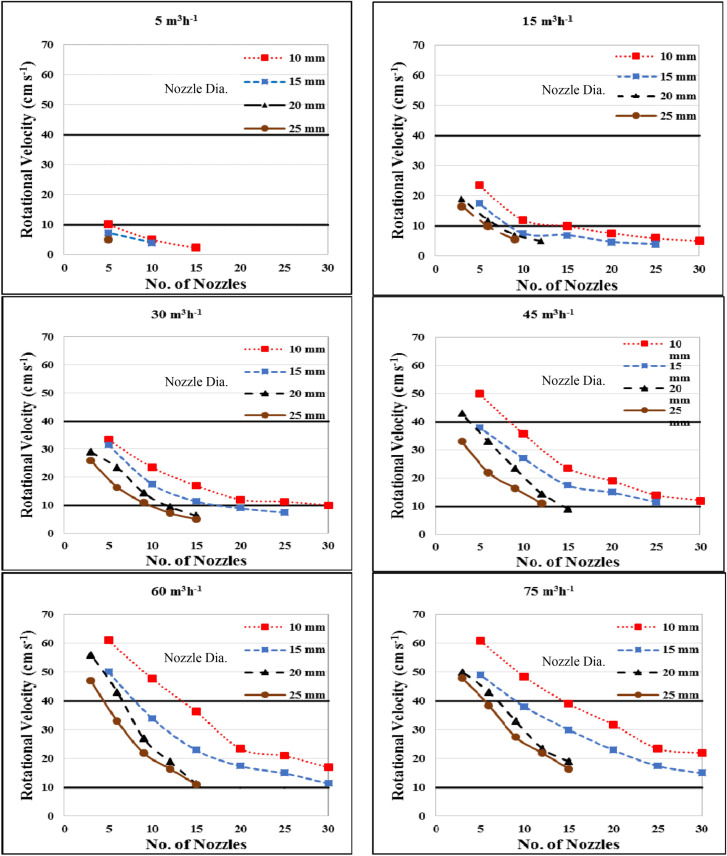
Effect of flow rate, nozzle diameter and number of nozzles on the rotational velocity of water in a circular tank.

Figure 3 shows the effect of flow rate, nozzle diameter and number of nozzles on the rotational velocity of water in a circular tank. The results indicate that the rotational velocity increases with increasing flow rates and deceasing nozzle diameter. It could be seen that, the rotational velocity decreased from 10.1 to 5.0 cm s^−1^, when the nozzle diameter increased from 10 to 20 mm, respectively for 5 nozzles used, and it decreased from 5.1 to 4.0 cm s^−1^, when the nozzle diameter increased from 10 to 15 mm, respectively, for 10 nozzles used with 5 m^3^ h^−1^ flow rate. At 15 m^3^ h^−1^, the rotational velocity was decreased from 23.5 to 17.5, 12.0 to 7.5, 10.0 to 6.9, 7.6 to 4.7 and 5.9 to 4.0 cm s^−1^ when the nozzle diameter increased from 10 to 20 mm, respectively, for 5, 10, 15, 20 and 25 nozzles, respectively. The results also indicate that when the nozzle diameter increased from 20 to 25 mm, the rotational velocity decreased from 19.0 to 16.5, 12.0 to 10.0 and 7.1 to 5.5 cm s^−1^ for 3, 6 and 9 nozzles, respectively, with 15 m^3^ h^−1^ flow rate.

At 30 m^3^ h^−1^ flow rate, the highest value of the rotational velocity was 33.5 cm s^−1^ was found for 5 nozzles and 10 mm nozzle diameter. While, the lowest value of the rotational velocity was 7.3 cm s^−1^ was found for 25 nozzles and 25 mm nozzle diameter. At 45 m^3^ h^−1^ flow rate, the rotational velocity ranged from 11.0 to 49.9 cm s^−1^ for all treatments under study.

At 60 m^3^ h^−1^ flow rate, the rotational velocity deceased from 61.0 to 50.1, 47.7 to 34.0, 36.3 to 23.0, 23.5 to 17.5, 21.0 to 15.0 and 17.0 to 11.5 cm s^−1^ when the nozzle diameter increased from 10 to 20 mm, respectively at 5, 10, 15, 20, 25 and 30 number of nozzles. The results also indicate that, when the nozzle diameter increased from 20 to 25 mm, the rotational velocity decreased from 56.0 to 47.0, 43.0 to 33.0, 27.0 to 22.0 and 19.0 to 16.5 cm s^−1^ at 3, 6, 9 and 12 nozzles, respectively.

At 75 m^3^ h^−1^ flow rate, the rotational velocity deceased from 60.9 to 49.1, 48.4 to 38.0, 39.0 to 30, 31.8 to 23.0, 23.5 to 17.5 and 22.0 to 15.0 cm s^−1^ when the nozzle diameter increased from 10 to 20 mm, respectively for 5, 10, 15, 20, 25 and 30 nozzles, respectively. The results also indicate that, when the nozzle diameter increased from 20 to 25 mm, the rotational velocity decreased from 50.48 to 43.0 to 38.5, 33.0 to 27.5 and 23.5 to 22.0 cm s^−1^ for 3, 6, 9 and 12 nozzles, respectively.

The results also indicate that the highest values of the rotational velocities were 10.1, 23.5, 33.5, 49.9, 60.9 and 61.0 cm s^−1^ were found for 5 nozzles and 10 mm nozzle diameter at 5, 15, 30, 45, 60 and 75 m^3^ h^−1^ flow rate, respectively. While, the lowest values of the rotational velocities were 4.0, 7.5 and 11.5 cm s^−1^ for 25 nozzles and 15 mm nozzle diameter at 5, 15 and 30 m^3^ h^−1^ flow rate, respectively. They were 11.5 and 15 cm s^−1^ were found for 30 nozzles and 15 mm nozzle diameter at 60 and 75 m^3^ h^−1^ flow rate, respectively. The velocity of water obtained seemed to be in the recommended range of safe and proper velocity for fish according to^12^. Due to it is effective compromise to allow heavy solids settle rapidly, yet sufficiently fast to create "good" hydraulics. Timmons and Youngs^18^ mentioned that the water velocity needed to maintain self-cleaning properties ranges from 3 to 40 cm s^−1^ varying greatly according to the physical properties of the biosolids. When fish swims at lower speed than its optimal, a large amount of energy will be used for higher spontaneous activity such as aggression. In contrast, when fish swim at higher speed than optimal, they become stressful, unstable, increase lactate production and fatigue^6^.

Multiple regression analysis was carried out to obtain a relationship between the rotational velocity of water as dependent variable and different both of flow rate and nozzle diameter as independent variables. The best fit for this relationship with coefficient of determination of 0.95 and an error of 1.06% is in the following form:-

3 $$ RV = 6.97 + 0.41Q - 0.19D\quad {\text{R}}^{{2}} = 0.95 $$

where RV is the rotational velocity of water, cm s^−1^, Q is the water flow rate, m^3^ h^−1^, D is the nozzle diameter, mm.

This equation could be applied in the range of 5 to 75 m^3^ h^−1^ water flow rate and from 10 to 25 mm of nozzle diameter.

### Impulse force of water

**Figure 4:**
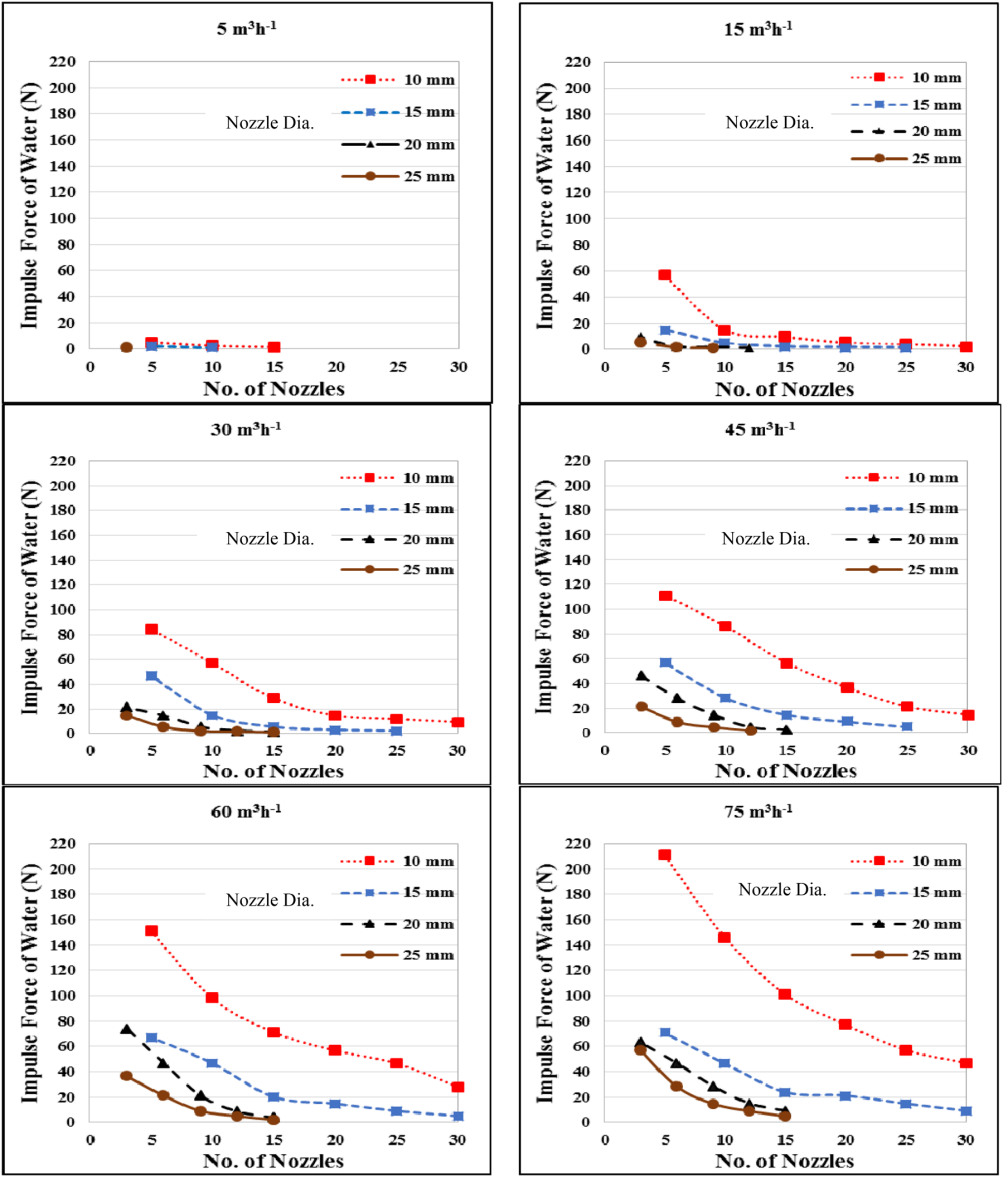
Effect of flow rate, nozzle diameter and number of nozzles on the impulse force of water in a circular tank.

Figure 4 shows the effect of flow rate, diameter and number of nozzles on the impulse force of water in a circular tank. The results indicate that the impulse force of water increases with increasing flow rates and deceasing nozzle diameter and number of nozzles. It could be seen that, the impulse force of water decreased from 5.1 to 1.7 N, when the number of nozzles increased from 5 to 15, respectively at 10 nozzle diameter, and it decreased from 2.3 to 1.2 N, when the number of nozzles increased from 5 to 10, respectively, at 15 diameter nozzle with 5 m^3^ h^−1^ flow rate. At 15 m^3^ h^−1^, the impulse force of water was decreased from 84.7 to 9.4 N when the number of nozzles increased from 5 to 30, respectively 10 mm diameter nozzle. The results also indicate that when the number of nozzles increased from 5 to 25, the impulse force of water decreased from 14.8 to 1.4 N at 15 mm nozzle diameter, respectively, and it decreased from 9.5 to 1.9 and 5.3 to 1.3 N at 20 and 25 mm, respectively, when the number of nozzles increased from 3 to 9.

At 30 m^3^ h^−1^ flow rate, the impulse force of water deceased from 84.7 to 46.9, 56.9 to 14.8, 28.5 to 5.3, 14.9 to 3.0 and 11.8 to 2.2 N when the nozzle diameter increased from 10 to 15 mm, respectively at 5, 10, 15, 20 and 25 nozzles. The results also indicate that, when the nozzle diameter increased from 20 to 25 mm, the impulse force of water decreased from 21.4 to 14.9, 14.8 to 5.4, 5.3 to 2.2 and 2.3 to 1.9 N for 3, 6, 9 and 12 nozzles, respectively.

At 45 m^3^ h^−1^ flow rate, the impulse force of water was ranged from 2.1 to 111.2 N for all treatments under this study. Also, at 60 m^3^ h^−1^ flow rate, the impulse force of water ranged from 5.1 to 151.3 N for all treatments under this study. At 75 m^3^ h^−1^ flow rate, the highest value of the impulse force of water 211.2 N was found for 5 numbers of nozzles and 10 mm nozzle diameter, respectively. While, the lowest value of the impulse force of water was 9.1 N was found for 12 nozzles and 25 mm nozzle diameter, respectively.

The results also indicate that the highest value of the impulse force of water 211.2 N was found for 5 nozzles and 10 mm nozzle diameter at 75 m^3^ h^−1^ flow rate, respectively. While, the lowest value of the impulse force of water was 1.2 N was found for 10 nozzles and 15 mm nozzle diameter at 5 m^3^ h^−1^ flow rate, respectively.

The results indicated that, the relationship between the rotational velocity and impulse force of water is linear relationship at the same treatments. When the rotational velocity increased from 10.7 to 37.6, 8.1 to 28.8, 10.2 to 36.0 and 11.0 to 31.9 cm s^−1^, the impulse force of water increased from 3.1 to 106.6, 1.8 to 31.1, 1.3 to 32.5 and 1.4 to 22.8 N, respectively, at the same treatments. The trend of these results agreed with those obtained by^19^.

Multiple regression analysis was carried out to obtain a relationship between the impulse force of water as dependent variable and different both of flow rate and nozzle diameter as independent variables. The best fit for this relationship with coefficient of determination of 0.88 and an error of 2.13% is in the following form:-

4 $$ F_{i} = 38.18 + 0.67Q - 2.35D\quad {\text{R}}^{{2}} = 0.88 $$

This equation could be applied in the range of 5 to 75 m^3^ h^−1^ water flow rate and from 10 to 25 mm of nozzle diameter.

### Average velocity of water

**Figure 5:**
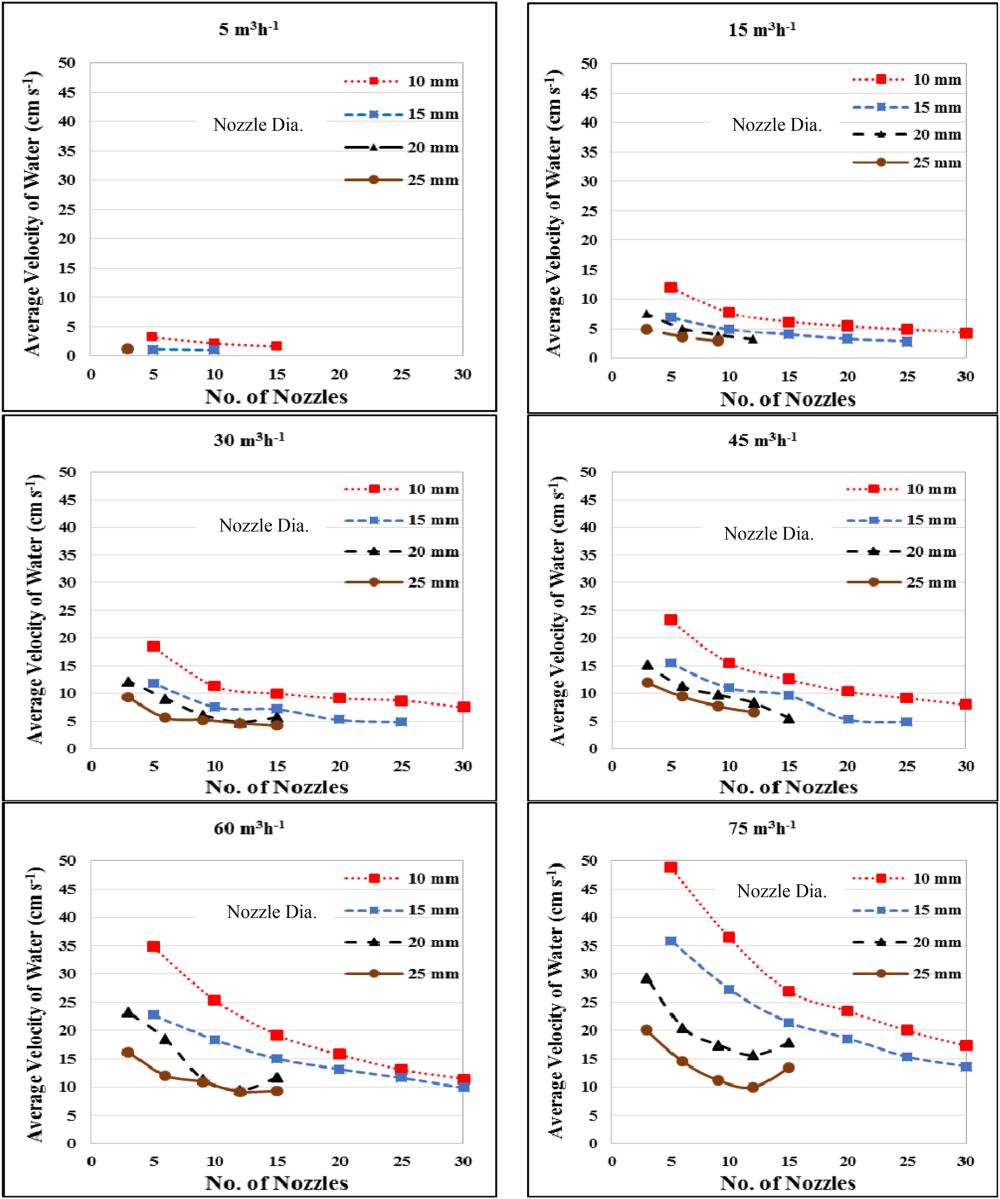
Effect of flow rate, nozzle diameter and number of nozzles on the average velocity of water in a circular tank.

Figure 5 shows the effect of flow rate, diameter and number of nozzles on the average velocity of water in a circular tank. The results indicate that the average velocity of water increases with increasing flow rates and deceasing nozzle diameter and number of nozzles. It could be seen that, the average velocity of water decreased from 3.32 to 1.59 cm s^−1^, when the number of nozzles increased from 5 to 15, respectively at 10 nozzle diameter, and it decreased from 1.13 to 1.07 cm s^−1^, when the number of nozzles increased from 5 to 10, respectively, at 15 diameter nozzle with 5 m^3^ h^−1^ flow rate. At 15 m^3^ h^−1^, the average velocity of water was decreased from 12.03 to 4.33 cm s^−1^ when the number of nozzles increased from 5 to 30, respectively 10 mm diameter nozzle. The results also indicate that when the number of nozzles increased from 5 to 25, the average velocity of water decreased from 6.93 to 2.89 cm s^−1^ at 15 mm nozzle diameter, respectively, and it decreased from 7.55 to 4.00 and 4.89 to 2.95 cm s^−1^ at 20 and 25 mm, respectively, when the number of nozzles increased from 3 to 9.

At 30 m^3^ h^−1^ flow rate, the highest value of the average velocity of water 18.51 cm s^−1^ was found for 5 nozzles and 10 mm nozzle diameter. While, the lowest value of the average velocity of water was 4.65 cm s^−1^ was found for 12 nozzles and 25 mm nozzle diameter. At 45 m^3^ h^−1^ flow rate, the average velocity of water ranged from 6.66 to 23.26 for all treatments under study, also, at 60 m^3^ h^−1^ flow rate, the average velocity of water ranged from 9.23 to 34.82 for all treatments under study. At 75 m^3^ h^−1^ flow rate, the average velocity of water ranged from 10.00 to 48.76 for all treatment of this study.

The results also indicate that the highest value of the average velocity of water 48.76 cm s^−1^ was found for 5 nozzles and 10 mm nozzle diameter at 75 m^3^ h^−1^ flow rate, respectively. While, the lowest value of the average velocity of water was 1.07 cm s^−1^ was found for 10 nozzles and 15 mm nozzle diameter at 5 m^3^ h^−1^ flow rate, respectively. These results agreed with those obtained by^18,20^. Fish distribution in the circular tank is influenced by the heterogeneity of water velocity in the area between inlet flow and the center of the tank^9^. Fish distribution in the circular tank is mostly concentrated in the area between high and low velocity area. The high velocity area will be avoided by most fishes as it requires high swimming energy, while dead volumes (low velocity area) are unfavorable condition for fish (low DO and higher metabolites accumulation)^21^.

Multiple regression analysis was carried out to obtain a relationship between the average velocity of water as dependent variable and different both of flow rate and nozzle diameter as independent variables. The best fit for this relationship with coefficient of determination of 0.91 and an error of 1.48% is in the following form:

5 $$ V_{avg} = 6.53 + 0.26Q - 0.37D\quad {\text{R}}^{{2}} = 0.91 $$

This equation could be applied in the range of 5 to 75 m^3^ h^−1^ water flow rate and from 10 to 25 mm of nozzle diameter.

## Conclusions

The experiment was carried out successively to determine the effect of flow rate, diameter and number of nozzles on the rotational velocity, impulse force and average velocity of water in fish circular tanks. The obtained results can be summarized as follows:

- The rotational velocity increases with increasing flow rates and decreasing diameters and numbers of nozzles. The highest values of the rotational velocities were 10.1, 23.5, 33.5, 49.9, 60.9 and 61.0 cm s^−1^ were found for 5 nozzles and 10 mm nozzle diameter at 5, 15, 30, 45, 60 and 75 m^3^ h^−1^ flow rate, respectively.
- The impulse force of water decreases with increasing flow rates and decreasing diameters and numbers of nozzles. The highest value of the impulse force of water 211.2 N was found for 5 nozzles and 10 mm nozzle diameter at 75 m^3^ h^−1^ flow rate, respectively. While, the lowest value of the impulse force of water was 1.2 N was found for 10 nozzles and 15 mm nozzle diameter at 5 m^3^ h^−1^ flow rate, respectively.
- The average velocity of water increases with increasing flow rates and decreasing diameters and numbers of nozzles. The highest value of the average velocity of water 48.76 cm s^−1^ was found for 5 nozzles and 10 mm nozzle diameter at 75 m^3^ h^−1^ flow rate, respectively. While, the lowest value of the average velocity of water was 1.07 cm s^−1^ was found for 10 nozzles and 15 mm nozzle diameter at 5 m^3^ h^−1^ flow rate, respectively.
- To obtain the rotational velocity in optimum range suitable for tilapia fish and for tank self-cleaning we recommended that the optimum flow rate is 30 m^3^ h^−1^ for 5 nozzles and 10 mm nozzle diameter.
- Further studies are recommended to be done on the effect of surface roughness on the rotational velocity, impulse force and average velocity of water in fish tanks.
- Theoretical studies should be done to optimize the factors affecting the rotational velocity, impulse force and average velocity of water in fish tanks.

## Data Availability

The datasets used and/or analyzed during the current study available from the corresponding author on reasonable request.
